# Acetylsalicylic Acid Suppresses Alcoholism-Induced Cognitive Impairment Associated with Atorvastatin Intake by Targeting Cerebral miRNA155 and NLRP3: In Vivo, and In Silico Study

**DOI:** 10.3390/pharmaceutics14030529

**Published:** 2022-02-27

**Authors:** Doaa I. Mohamed, Dalia Alaa El-Din Aly El-Waseef, Enas S. Nabih, Omnyah A. El-Kharashi, Hanaa F. Abd El-Kareem, Hebatallah H. Abo Nahas, Basel A. Abdel-Wahab, Yosra A. Helmy, Samar Zuhair Alshawwa, Essa M. Saied

**Affiliations:** 1 Department of Clinical Pharmacology and Therapeutics, Faculty of Medicine, Ain Shams University, Cairo 11566, Egypt; omnyah2011@gmail.com; 2 Department of Histology and Cell Biology, Faculty of Medicine, Ain Shams University, Cairo 11566, Egypt; daliaalaaelwaseef@gmail.com; 3 Department of Medical Biochemistry and Molecular Biology, Faculty of Medicine, Ain Shams University, Cairo 11566, Egypt; enassamer@hotmail.com; 4 Zoology Department, Faculty of Science, Ain Shams University, Abbasseya, Cairo 11566, Egypt; hanaafathy@sci.asu.edu.eg; 5 Zoology Department, Faculty of Science, Suez Canal University, Ismailia 41522, Egypt; hebatallah_hassan@science.suez.edu.eg; 6 Department of Medical Pharmacology, College of Medicine, Assiut University, Assiut 71111, Egypt; basel_post@msn.com; 7 Department of Pharmacology, College of Pharmacy, Najran University, Najran 1988, Saudi Arabia; 8 Department of Veterinary Science, College of Agriculture, Food, and Environment, University of Kentucky, Lexington, KY 40503, USA; yosra.helmy@uky.edu; 9 Department of Animal Hygiene, Zoonoses and Animal Ethology, Faculty of Veterinary Medicine, Suez Canal University, Ismailia 41522, Egypt; 10 Department of Pharmaceutical Sciences, College of Pharmacy, Princess Nourah bint Abdulrahman University, P.O. Box 84428, Riyadh 11671, Saudi Arabia; szalshawwa@pnu.edu.sa; 11 Chemistry Department, Faculty of Science, Suez Canal University, Ismailia 41522, Egypt; 12 Institute for Chemistry, Humboldt Universität zu Berlin, Brook-Taylor-Str. 2, 12489 Berlin, Germany

**Keywords:** alcoholism, statins, atorvastatin, acetylsalicylic acid, histopathology, miRNA155, NLRP3 inflammasomes, molecular docking

## Abstract

Alcoholism is one of the most common diseases that can lead to the development of several chronic diseases including steatosis, and cognitive dysfunction. Statins are lipid-lowering drugs that are commonly prescribed for patients with fatty liver diseases; however, the exact effect of statins on cognitive function is still not fully understood. In the present study, we have investigated the molecular and microscopic basis of cognitive impairment induced by alcohol and/or Atorvastatin (ATOR) administration to male Wistar albino rats and explored the possible protective effect of acetylsalicylic acid (ASA). The biochemical analysis indicated that either alcohol or ATOR or together in combination produced a significant increase in the nucleotide-binding domain–like receptor 3 (NLRP3), interleukin-1β (IL-1β) miRNA155 expression levels in the frontal cortex of the brain tissue. The histological and morphometric analysis showed signs of degeneration in the neurons and the glial cells with aggregations of inflammatory cells and a decrease in the mean thickness of the frontal cortex. Immunohistochemical analysis showed a significant increase in the caspase-8 immunoreaction in the neurons and glial cells of the frontal cortex. Interestingly, administration of ASA reversed the deleterious effect of the alcohol and ATOR intake and improved the cognitive function as indicated by biochemical and histological analysis. ASA significantly decreased the expression levels of miRNA155, NLRP3, and IL1B, and produced a significant decrease in caspase-8 immunoreaction in the neurons and glial cells of the frontal cortex with a reduction in the process of neuroinflammation and neuronal damage. To further investigate these findings, we have performed an extensive molecular docking study to investigate the binding affinity of ASA to the binding pockets of the NLRP3 protein. Our results indicated that ASA has high binding scores toward the active sites of the NLRP3 NACHT domain with the ability to bind to the NLRP3 pockets by a set of hydrophilic and hydrophobic interactions. Taken together, the present study highlights the protective pharmacological effect of ASA to attenuate the deleterious effect of alcohol intake and long term ATOR therapy on the cognitive function via targeting miRNA155 and NLRP3 proteins.

## 1. Introduction

With over 2.5 million deaths annually worldwide, alcohol abuse causes a myriad of serious health consequences leading to physical and mental damage [1]. Alcohol is absorbed in the gastrointestinal tract, and transferred to the liver and lungs where it is metabolized leading to direct toxicity, an accumulation of fatty acid, ethyl esters and oxidative stress [2]. Furthermore, alcohol can impact blood pressure giving rise to cardiovascular disease, coronary heart disease, stroke, peripheral arterial disease, and cardiomyopathy [3]. Chronic alcohol abuse might affect the lung parenchyma increasing the incidence of acute respiratory disease [4]. Alcoholic fatty liver (steatosis), alcoholic hepatitis, and fibrosis are associated with the progression of liver cirrhosis, with the highest risk in alcoholism. Chronic alcoholism is also implicated with impaired cognitive function and structural abnormalities in different brain areas [5]. Long-term alcohol intake results in neuroinflammation and neurodegeneration in humans as well as animal models [6]. Several studies reported that the prefrontal cortex, which plays a vital role in cognitive functions, is more vulnerable to alcohol-related brain damage [7], while steatosis occurs in almost all individuals who excessively consume alcohol. Statins are lipid-lowering drugs that act as competitive inhibitors for 3-hydroxy-3-methylglutaryl coenzyme A reductase (HMG-CoA), a key enzyme in the synthesis of cholesterol. Statins are the first drug line for treating lipid disorders and are commonly prescribed for patients with alcoholic and non-alcoholic fatty liver diseases to help in the reduction of atherosclerotic cardiovascular events by reducing low-density lipoprotein cholesterol [8]. They are also recommended for liver cirrhosis to reduce disease complications and the mortality rate [9]. Although statins are prevalent drugs, they have several reported side effects such as, muscle complaints, neurological and neurocognitive effects, renal toxicity, and hepatotoxicity [10]. Among the known statins, Atorvastatin (ATOR), a synthetic lipophilic statin, can passively diffuse across the cell membrane which decreases its hepatoselectivity [10].

Yang et al. recently reported that chronic alcohol intake upregulates the expression of proinflammatory interleukin (IL)-1β cytokine (IL-1β) and nucleotide-binding domain–like receptor3 (NLRP3) in different brain areas [11]. NLRP3 is a subcellular multiprotein complex that is expressed in the central nervous system (CNS) and it is responsible for neuroinflammation and associated brain disease. NLRP3 is an inflammasome-forming NLR that has been involved in the pathogenesis of many inflammatory diseases [12]. The NLRP3 inflammasome is a critical component of the innate immune system that mediates caspase-1 activation and the secretion of proinflammatory cytokines: IL-1β and IL-18 in response to cellular damage [13]. Activation of NLRP3 inflammasomes by caspase-8, an apoptotic caspase, plays a crucial role in regulating the inflammasome activation and pro-inflammatory cytokine levels [13]. Further, stimulation of NLRP3 causes the induction of the apoptosis-associated speck-like protein containing a C-terminal caspase recruitment domain (ASC), which activates the cleavage of pro-caspase-1 into its cleaved form, caspase-1, which in consequence plays a role in the maturation of IL-1β into its active form [14]. Recently, it was shown that caspase-8 modulates the NLRP3-dependent caspase-1 signaling cascades that initiate IL-1β production and pyroptotic cell death [15].

Cognitive dysfunction has been considered as a potential severe consequence of alcohol abuse. Alcohol exposure induces the dysregulation of microRNAs (miRNAs) of the brain tissues. In human post-mortem brain samples, robust changes in miRNA expression have been reported in the prefrontal cortex of subjects with a history of chronic alcohol abuse [16,17]. Among the different brain miRNAs, miRNA-155 is the most prominent miRNA that is significantly upregulated in the brain of chronic alcoholism with possible implications for inflammatory conditions and cognitive dysfunction [18,19]. miRNA155 is a proinflammatory intermediary of the CNS that is stimulated within macrophages and microglia [20]. In an interesting study, Lippai et al. showed that alcohol abuse stimulates neuroinflammation in the cerebellum by mediating miR-155 in a TLR4-dependent manner [21]. Further, it was shown that miRNA155 enhances the expression of NLRP3-IL-1β which has been involved in the pathogenesis of alcohol-induced neuroinflammation [21]. Interestingly, several studies have demonstrated that ATOR can also affect the NLRP3 inflammasomes and IL-1β expression [22,23,24]. Indeed, the effect of ATOR (statins) on cognitive function has not been fully investigated. It is well known that statins induce the suppression of cholesterol synthesis which could cause cognitive impairment by affecting cholesterol levels locally in the CNS [25]. Although the association of miRNA155 and NLRP3 to cognitive function in alcoholism has been recently explored, the correlation between the miRNA155–NLRP3 pathway and alcohol-induced cognitive dysfunction associated with ATOR treatment is still not fully understood.

Acetylsalicylic acid (ASA, aspirin) is a non-steroidal anti-inflammatory drug that is usually used as an analgesic, anti-pyretic, and anti-inflammatory drug. With numerous benefits over other non-steroidal drugs, ASA decreases the risk of CVD by decreasing blood clot formation and inhibiting platelets’ activation through its antithrombotic properties [26]. Preclinical models have suggested that ASA may decrease neuroinflammation and oxidative stress in the CNS. The pleiotropic mechanisms of action of ASA could aid in the prevention of cognitive dementia [27]. Recent studies showed that a low dose of ASA alleviates the hepatotoxic effects of alcohol by modulating the NLRP3 inflammasome pathway and IL-1β [28,29]. Further studies have showed that ASA improves the endothelial dysfunction by targeting NLRP3 inflammasome through the ROS/TXNIP pathway [26]. These results suggest that ASA could be applied for the treatment of cognitive dysfunction by targeting the NLRP3-IL-1β pathway.

Encouraged by the above-mentioned facts, the presented study aimed at exploring the role of the miRNA155–NLRP3 inflammasomes pathway on the cognitive dysfunction induced by chronic alcohol ingestion and/or ATOR treatment. Further, we investigated the possible role of ASA in targeting this pathway via biochemical, histopathological, immunohistochemical, and in silico molecular modelling studies.

## 2. Materials and Methods

### 2.1. Animals and Grouping

Animal experiments have been approved by the Institutional Animal Ethics Committee for Ain Shams University, Faculty of Medicine (No. FWA 00021368, 5/2021). Thirty Wistar male rats (weight 150–200 g) have been acquired from the National Research Institute (Cairo, Egypt). The animals were housed with lighting control (12 h light/dark cycle) at 22 °C. An adaptation period of 1 week was allowed before admission of the experimental procedure. The rats have been randomly and equally divided into 5 groups (*n* = 6):

Group I; The control group.
Group II; Chronic alcohol ingestion (10 g/kg/day) [30].
Group III; Atorvastatin (10 mg/kg/day, p.o.) treated [31].
Group IV; Chronic alcohol ingestion + atorvastatin.
Group V; Chronic alcohol ingestion + atorvastatin + ASA (10 mg/kg/day, p.o.) [32].

### 2.2. Chemical Reagents and Drugs

Absolute ethanol (100%), atorvastatin and ASA have been acquired from the Sigma Chemical Company, Cairo, Egypt [30,33,34].

### 2.3. Induction of Alcoholic Brain Injury

After acclimation for 6-7 days, the animals were treated with alcohol (10 g/kg; oral gavage) as a water solution (with solutions maximally containing 56% alcohol) for 10 weeks and treated with atorvastatin (10 mg/kg) alone and/or in combination with a dose of ASA (10 mg/kg) for the same duration. All rats had frequent standard rat chow available during the 6-week period [28].

### 2.4. Cognitive Function Assessment; Morris Water Maze (MWM)

The Morris water maze (diameter 1.8 m) was used for testing, along with a "Atlantis platform" (diameter 10 cm). The platform was placed in the middle of the pool's northeast quadrant, the water was made dim with powdered milk, and the chamber was lighted by four 30-W spotlights pointing at the ceiling. The water was kept at a constant temperature of 23 °C. Numerous constants, or prominent visual signals, were present in the testing room (posters, objects, and equipment). A video camera was installed just above the pool on the ceiling to monitor each rat's swim path. Each day for five days, rats underwent four trials in the Morris water-maze, without any signals indicating the position of the platform. The submerged platform remained constant in one quadrant of the maze throughout testing, and the latency to locate it (as well as the distance travelled by the rats) were recorded. Each trial in this and subsequent studies began with the precise placement of an individual rat into the water, facing the pools outside edge, at one of four possible beginning places (e.g., north, south, east, west). Each experiment began at a random site, with the proviso that all start locations were used on any given day. A trial was ended, and the latency was measured when the rat reached and remained on the platform for ten seconds. The experiment was cancelled if the rat did not reach the platform within 120 seconds, and the rat was placed on the platform for 10 seconds. Following that, rats were transported to a dry holding cage for 60 seconds until the following session. Rats were moved to their home cages following training. On the sixth day, rats received an additional 60-second probe session in which the pool lacked a platform. As before, rats were placed in the pool and the latency to reach the target quadrant as well as the duration spent within the quadrant of the platform were recorded [35].

### 2.5. Biochemical Analysis

Stored brain samples (from frontal lobes) at −80 °C were used in the determination of the miRNA155, NLRP3 and IL-1β expression level.

#### Real-Time PCR

Total RNA was extracted utilizing a RNeasy Mini Kit. The RNA was reversed transcribed utilizing a QuantiTect Reverse Transcription Kit and real-time PCR was performed for the miRNA155 NLRP3 and IL-1β using a QuantiTect SYBR Green PCR Kit. The kits were acquired from Qiagen, Hilden, Germany. β-actin has been utilized as the housekeeping gene. The miRNA155 primers were Forward: 5′-AGGGAAATCGTGCGTGAC-3′, Reverse: 5′-CGCTCATTGCCGATAGTG-3′ (GenBank NM_031144.3). For the NLRP3, the primers were Forward: 5′−CCAGGGCTCTGTTCATTG-3′, Reverse: 5-CCTTGGCTTTCACTTCG-3′ (GenBank NM_001191642.1) and for the IL1β, the primers were Forward: 5′-CACCTTCTTTTCCTTCATCTTTG-3′, Reverse: 5′-GTCGTTGCTTGTCTCTCCTTGTA-3′ (GenBank NM_031512.2). The PCR reaction mixture was 20 μL and contained 500 ng RT product and 0.5 μM of each primer. The cycling technique included initial heating for 15 min at 95 °C followed by 40 cycles. Each cycle consisted of a denaturation stage for 15 s at 94 °C, annealing step for 30 s at 55 °C, and extension step for 30 s at 70 °C. The relative expression of target genes was estimated using the 2^−ΔΔCt^ equation.

### 2.6. Histology Study

The frontal lobe of the brain was extracted immediately at the end of the experiment and divided into two halves for the following analysis:

- Light microscopic (LM) analysis

Half of the frontal lobe was divided into small pieces and immersed in 10% of neutral buffered formalin followed by dehydration to get paraffin blocks for the LM analysis. The paraffin blocks have been divided into sections with 5 micron-thick for a H&E stain. Immunohistochemical staining has been performed employing an avidin biotin-peroxidase method for the detection of cleaved caspase-8 (acquired from Cell Signaling Technology, Danvers, MA, USA). The reaction was performed with a DAB solution (acquired from DAKO, Glostrup, Denmark) for 10 min. Subsequently, the counterstain was performed utilizing Mayer’s hematoxylin. The sections for negative control were obtained following the same protocol, with the exception of the use of the primary antibody [36].

ii. Semi-thin sections analysis

Examining semi-thin sections—stained with toluidine blue—was a very important step before preparing ultrathin sections to be examined by a transmission electron microscope (TEM). The semi-thin sections helped in examining a large field of the specimen with a good resolution using a light microscope. This was important to locate and trim the specific area in the specimen that was required to be later examined by the TEM. This is especially relevant in brain samples where it is very important to determine the brain region that will be studied. In addition, the high resolution of the brain semi-thin sections helped in differentiating between the neurons and glial cells by their morphology and the specific criteria of their nuclei. This was based on an algorithm that can be used to systematically distinguish the cellular types in the cerebral cortex, using semi-thin and ultrathin brain sections [37].

iii. Transmission electron microscopic (TEM) analysis

The half of the frontal lobe material was chopped into small (1 mm^3^) pieces and preserved in 2.5% glutaraldehyde for TEM analysis. Subsequently, we cut semi-thin sections (1 µm) and stained them with toluidine blue stain. Ultra-sections as thin as 80 nm were stained with lead citrate and uranyl acetate before being analyzed by TEM (Joel, Tokyo, Japan) at the EM unit, Ain Shams University, Faculty of Science [38].

### 2.7. Morphometric Study

The image analyzer computer system, Leica Qwin 500, UK at the Faculty of Medicine, Ain Shams University, Department of Histology and Cell Biology, was used to evaluate the cerebral cortex thickness (frontal cortex) (×20 power lens) and the area % of Caspase-8 positive reaction in the cells of the cerebral cortex (×40 power lens). Each parameter was assessed in 5 fields of two serial sections in each group for all animals.

### 2.8. In Silico Molecular Modelling Study

The binding affinity of ASA was explored toward the active site of the NLRP3 protein by in silico molecular docking using Molecular Operating Environment software (MOE^®^, 2015.10). In the protein data bank (PDB), there was only two crystal structures available for the NLRP3 domain in complex with a substrate and/or 2-furanylsulfonyl-urea derivative inhibitor (PDB code: *7alv* and *6npy*) [39,40]. To deeply investigate the potency of ASA as an inhibitor for the NLRP3 protein, we have explored their binding toward the different available 3D crystal structures of the NLRP3 protein. The 3D structures of the NLRP3 (PDB code: *7alv* and *6npy*) were retrieved from PDB (http://www.rcsb.org/pdb, 6 February 2022). The structures of the ASA were obtained in 2D using the ChemDraw program. The 3D structures of the ASA were obtained as MDB files employing the Discovery Studio software. The extra chains and water molecules were deleted. The 3D protonated protein structure was obtained employing the default protocol in the MOE program. Energy minimization, expression of partial charges, and geometry optimizations were obtained by applying the default Conf Search module and MMFF94x force field in the MOE program. The docking protocol was applied using the London dG scoring function and Triangle Matcher placement method as reported previously [41,42,43,44,45,46]. The docking parameters and protocol were then validated by redocking the co-crystallized ligand into the active site of the protein. The obtained binding poses were then compared to that of the reported crystal structure to affirm the similarity in binding mode. The validated protocol was then used to dock the ASA to the NLRP3 binding site to explore their binding affinity. The acquired results were collected and assessed to obtain the poses with the highest protein-ligand binding scores.

### 2.9. Statistical Analysis

Sample size was defined utilizing the GraphPad StatMate software program, 16 January 1998 Version 1. Statistical analysis was employed utilizing the GraphPad Prism software program, version 5.0 (2007) (Inc., San Diego, CA, USA) and Excel (2007). Statistical difference among the groups has been defined employing one-way (ANOVA) analysis, followed by a post hoc Tukey test to compare between more than two groups.

## 3. Results

### 3.1. Effect of Alcohol and/or ATOR Treatment on Cognitive Function and Assessment of ASA Administration

We first examined the effect of alcohol administration and ATOR intake on cognitive function by using the Morris water maze test. As shown in Table 1, the administration of alcohol and/or ATOR induced a significant increase in latency time to reach the target quadrant in 1–6 days, while a significant decrease was observed in the percentage of time spent in the target quadrant during the sixth day. These results could be attributed to the direct toxic effect of alcohol on the brain and through the release of various mediators such as an inflammatory, endogenous antioxidant, caspases and brain-derived neurotrophic factor BDNF, which mainly affects the prefrontal area. The ATOR treatment can also potentiate the neuroinflammatory process via the activation of NLRP3 and its subsequent mediator IL1B-inducing cognitive impairment. Interestingly, the ASA treatment decreased the latency time to reach the target quadrant in the six days and significantly decreased the percentage time spent in the target quadrant on the sixth day (*p* < 0.001), when compared to the alcohol and/or ATOR groups. These results indicate that ASA with its nonsteroidal anti-inflammatory effect could repress the production of pro-inflammatory molecules and accordingly decrease the neuro-inflammation and oxidative stress in the CNS. This pleiotropic mode of action of the ASA could aid in the prevention of cognitive decline in rats treated with alcohol and/or ATOR.

### 3.2. Biochemical Analysis

#### 3.2.1. Effect of Alcohol and/or ATOR Treatment on miRNA155 Expression and Influence of ASA Administration

**Figure 1:**
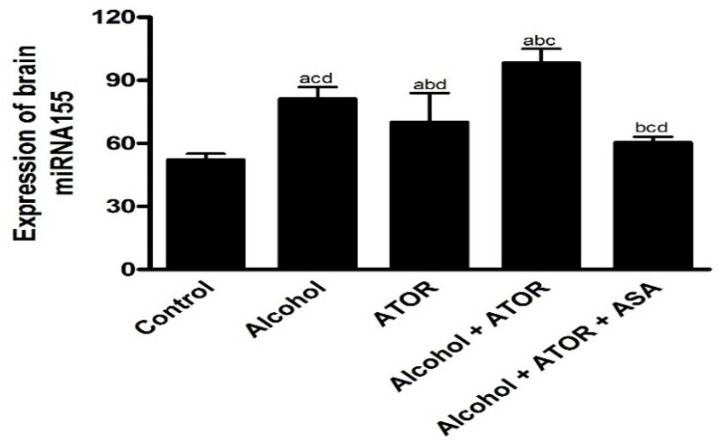
Expression of brain miRNA155 in the different treated groups (mean ± SD). Data are mean ± SD of 6 rats per group. *p* < 0.05 is significant; ^a^ *p* versus control group, ^b^ *p* versus Alcohol group, ^c^ *p* versus ATOR group, ^d^ *p* versus Alcohol + ATOR + ASA group.

Dysregulation of brain miRNAs has been recently correlated to alcohol exposure. The upregulation of miRNA155 expression in brain tissue was found to induce neuroinflammation via amplification of IL-1β, IL-6 and TNF-α with an implication on cognitive dysfunction; however, the correlation between miRNA155 and alcohol-induced cognitive impairment associated with an ATOR treatment is still elusive. To gain insights into this correlation and the molecular changes related to the brain neuroinflammation process, miRNA155 expression in the prefrontal cortex was evaluated. As shown in Figure 1, alcohol and/or ATOR administration significantly increased (*p* < 0.0001) the brain miRNA155 expression as compared to the control group. These findings indicate that alcohol and/or ATOR treatments have a direct effect on upregulating miRNA155 expression which plays an important role in neuroinflammation in activated microglia and macrophages. The high expression of miRNA155 induces the release of inflammatory mediators, including nitric oxide, and pro-inflammatory cytokines (IL-1β, TNFα), and increases microglial cell-mediated neurotoxicity. Furthermore, miR-155 has been shown to be involved in chemokine signaling, both in the periphery and within the CNS.

On the other hand, the ASA-treated group showed a significant decrease (*p* < 0.0001) in brain miRNA155 expression, indicating the ability of ASA to reduce the possible neuroinflammation induced by miRNA155 overexpression that occurred due to the alcohol and/or ATOR administration. These results could be attributed to the ASA’s anti-inflammatory properties and its ability to downregulate TNF-α-and NF-κB-dependent miR-155 transcriptional biogenesis.

#### 3.2.2. Effect of ASA on Brain NLRP3 and IL-1β Expression after Alcohol and/or ATOR Treatment

**Figure 2:**
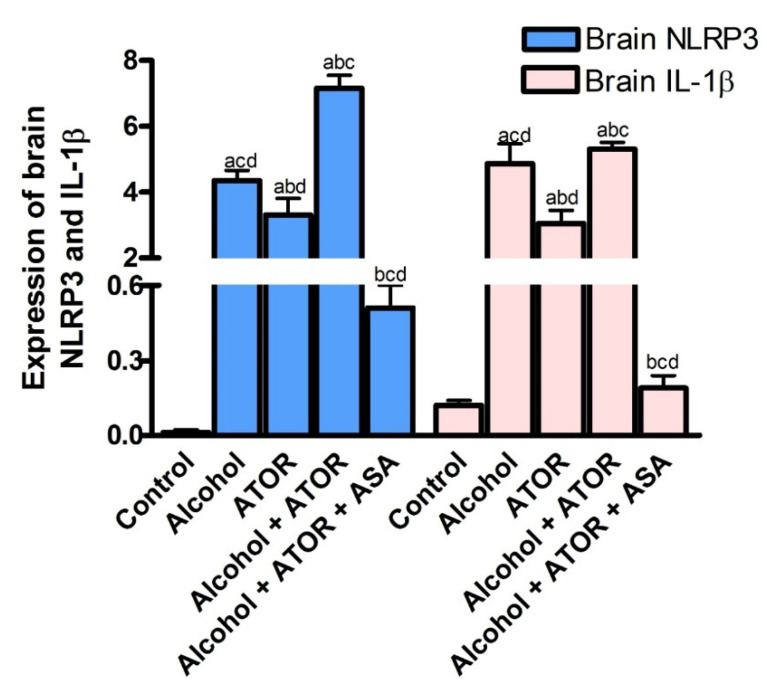
Effect of alcohol and/or ATOR administration on the expression of brain NLRP3, and IL-1β and assessment of ASA treatment (mean ± SD). Data are mean ± SD of 6 rats per group. *p* < 0.05 is significant; ^a^ *p* versus control group, ^b^ *p* versus Alcohol group, ^c^ *p* versus ATOR group, ^d^ *p* versus Alcohol + ATOR + ASA group.

Neuroinflammation is a known factor in the pathogenesis of neurodegenerative diseases and impairs the cognition and memory function. To examine the process of neuroinflammation, the expression of NLRP3 and IL-1β were examined. As depicted in Figure 2, the administration of alcohol and/or ATOR significantly increased (*p* < 0.0001) NLRP3 and IL1 β expression. These results reveal the possible role of NLRP3 inflammasome in promoting neuroinflammation and aggravating cognitive impairment upon alcohol and/or ATOR intake. NLRP3 inflammasome has been known as a key contributor to promote the secretion of pro-inflammatory IL-1β and the formation of caspase-1, which leads to aggravating the inflammatory reaction, neutrophil infiltration, neurotoxicity and a worsening neurological function.

The ASA treatment caused a significant decrease (*p* < 0.0001) in the NLRP3 and IL-1β brain expression and was able to ameliorate the process of neuroinflammation and consequently the cognitive impairment induced by the administration of alcohol and/or ATOR. This could be explained by the ability of the ASA to downregulate pro-IL-1β and pro-IL-18 transcription and to stimulate the brain autophagy leading to elimination of impaired brain mitochondria and reactive oxygen species, which finally leads to the amelioration of neuroinflammation and cognitive decline. Overall, ASA could induce high epigenetic regulation of the inflammasome pathway by targeting miRNA 155 and downregulating NLRP3 and IL1 β expression in the brain tissue (Figure 2).

### 3.3. Histological Results

#### 3.3.1. Light Microscopic Analysis

**Figure 3:**
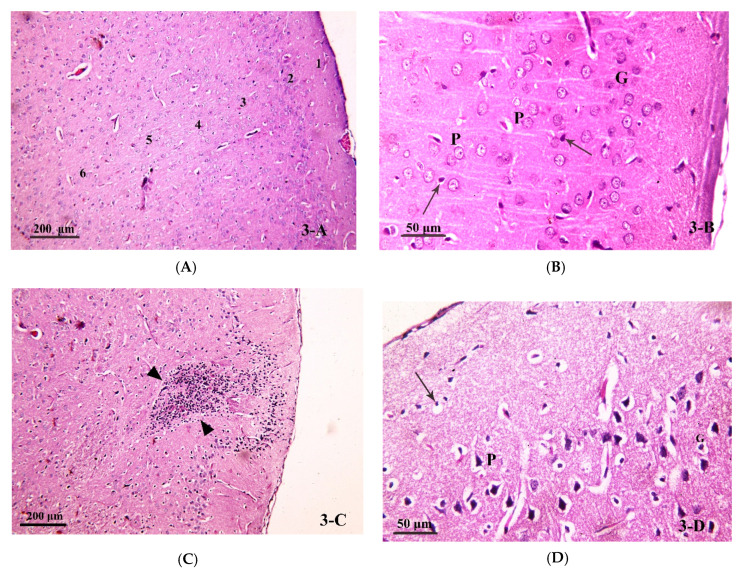

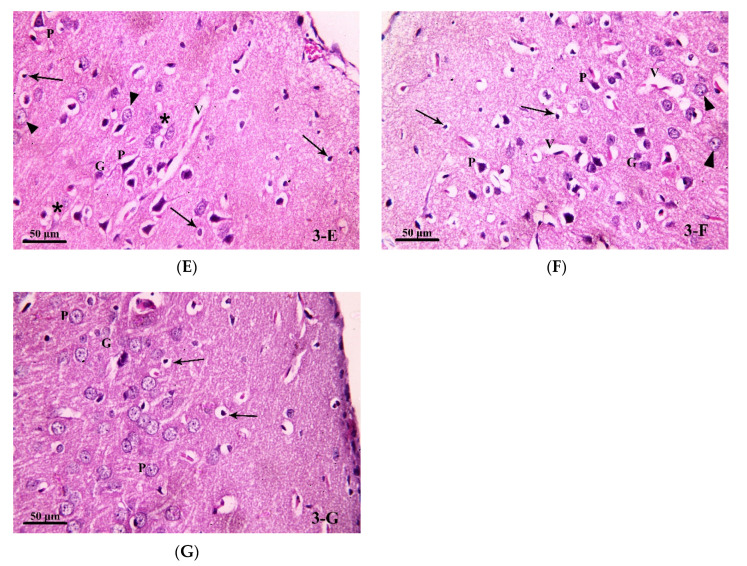
Photomicrograph of brain tissue (H& E stain) from (**A**,**B**) Group I, showing the six layers of the cerebral cortex in the control group (H&E × 100). The nerve cells (pyramidal (P) and granule (G)) in the different layers of the cerebral cortex with large, rounded nuclei and prominent nucleoli. Nerve cells are separated by homogenous neuropil, containing small, deeply stained nuclei—most probably glial cells—(↑) (H&E × 400). (**C**,**D**) Group II showed distorted layers of the cerebral cortex in the alcoholic group. An area of aggregated mononuclear inflammatory cells (granuloma) can be seen (▲) (H&E × 100). Most of the pyramidal cells (P) and granule cells (G) in the different layers seen are shrunken, condensed, deeply stained and surrounded by a hallow. Small, deeply stained nuclei—most probably glial cells—are also surrounded by hallows (↑). Notice the pale, vacuolated neuropil (*) and the dilated blood vessel (V) (H&E × 400). (**E**) Group III, some pyramidal cells (P) and granule cells (G) are seen shrunken, condensed, deeply stained and surrounded by hallows. Notice some nerve cells have large, rounded nuclei and prominent nucleoli (▲). Small, deeply stained nuclei—most probably glial cells—are also surrounded by hallows (↑). The neuropil is pale and vacuolated (*). A dilated blood vessel (V) is seen (H&E × 400). (**F**) Group IV showed distorted layers of the cerebral cortex in group IV. Many pyramidal cells (P) and granule cells (G) are seen shrunken, condensed, deeply stained and surrounded by hallows. Notice few nerve cells have large, rounded nuclei and prominent nucleoli (▲). Small, deeply stained nuclei—most probably glial cells—are also surrounded by hallows (↑). Dilated blood vessels (V) are seen (H&E × 400). (**G**) Group V showed a more regular arrangement of layers of the cerebral cortex in group V. Most of the pyramidal(P) and granule (G) cells have large, rounded nuclei. Some nuclei have prominent nucleoli and others are vacuolated (∆). Some small, deeply stained nuclei—most probably glial cells—are surrounded by hallows (↑) (H&E × 400).

To affirm our biochemical results, the effect of chronic alcohol and/or ATOR intake on brain tissue and the possible protective effect of ASA was implemented using light microscopic examination of H&E stained brain sections. Light microscopic examination of the control group (Group I) showed that the frontal cortex was formed of six layers with no sharp demarcations; molecular, outer granular, outer pyramidal, inner granular, inner pyramidal and multiform (Figure 3A). These layers contained nerve cells (mainly pyramidal cells and granule cells) and glial cells. Nerve cells (pyramidal (P) and granule (G)) in the different layers of the cerebral cortex appeared as large cells with large, rounded nuclei and prominent nucleoli. The nerve cells were separated by homogenous neuropil, containing small, deeply stained nuclei—most probably glial cells—(Figure 3B). The alcohol-treated group (Group II) showed distortion of the layers of the cerebral cortex. Occasionally, areas of aggregated mononuclear inflammatory cells (granuloma) were seen (Figure 3C). Most of the pyramidal cells and granule cells in the different layers were shrunken, condensed, and deeply stained and surrounded by a hallow. Small, deeply stained nuclei—most probably glial cells—were also surrounded by hallows. The neuropil was pale and vacuolated, and the blood vessels were dilated (Figure 3D). In the ATOR-treated group (Group III), some pyramidal cells and granule cells were shrunken, condensed, deeply stained and surrounded by hallows; however, some nerve cells had large, rounded nuclei and prominent nucleoli. Small, deeply stained nuclei, most probably glial cells, were also surrounded by hallows. The neuropil was pale and vacuolated. Some dilated blood vessels were seen (Figure 3E). The alcohol-group treated with ATOR (Group IV) showed distorted layers of the cerebral cortex. Many pyramidal cells and granule cells were shrunken, condensed, deeply stained and surrounded by hallows. Few nerve cells had large, rounded nuclei and prominent nucleoli. Small, deeply stained nuclei—most probably glial cells—were also surrounded by hallows. The neuropil was pale and markedly vacuolated. Dilated blood vessels were seen (Figure 3F). Finally, the alcohol-group treated with both the ATOR and ASA (Group V) showed more regular arrangement of the layers of the cerebral cortex. Most of the pyramidal and granule cells had large, rounded nuclei. Some nuclei had prominent nucleoli and others were vacuolated. Some small, deeply stained nuclei, most probably glial cells, were surrounded by hallows. The neuropil appeared homogenous and few dilated blood vessels were detected (Figure 3G).

Moreover, we have investigated the thickness of the frontal cortex. A morphometric study showed a significant decrease in the mean thickness of the frontal cortex in the alcohol-group (Group II) as compared to the control group (Group I), ATOR-treated group (Group III) and alcohol-group treated with ATOR + ASA (Group V). While it showed non-significant change as compared to the alcohol-group treated with ATOR (Group IV). On the other hand, Group V showed a significant increase as compared to Group II, Group III and Group IV. Moreover, there was a non-significant change when compared to Group V to Group I, as well as for Group II, Group III and Group IV when compared to each other (Table 2). These results indicate that there was damage that occurred in the prefrontal cortex due to chronic alcohol intake associated with the ATOR administration. While the treatment with the ASA improved these damages.

#### 3.3.2. Caspase-8 Immunostained Analysis

**Figure 4:**
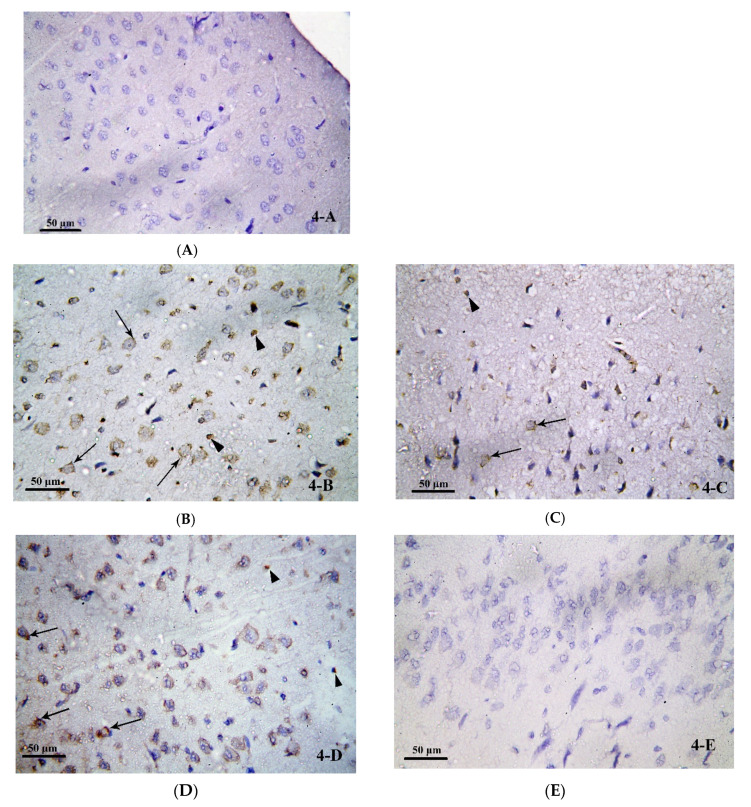
Photomicrograph of brain tissue from (**A**) Group I, showing a negative caspase-8 immune-reaction in nerve cells and glial cells in the frontal cortex of the control group. (**B**) Group II showed positive immune-reaction to caspase-8 in the cytoplasm of some nerve cells (↑) and glial cells (▲). (**C**) Group III showed a positive immune-reaction to caspase-8 in the cytoplasm of some nerve cells (↑) and glial cells (▲). (**D**) Group IV, showing a positive immune-reaction to caspase-8 (↑) in the cytoplasm of some nerve cells and glial cells. (**E**) Group V showed a negative caspase-8 immune-reactions in nerve cells and glial cells in the frontal cortex of group V. (Avidin Biotin Peroxidase for Caspase-8 × 400).

Caspase-8 is considered as a pro-apoptotic initiator which mediates the production of IL-1β and hence neuroinflammation. In order to investigate the role of caspase-8 in the process of neuroinflammation under treatment with alcohol and/or ATOR, and the possible protective role of ASA, caspase-8 immunostained analysis on the brain tissue was performed. A negative caspase-8 immune-reaction was detected in the nerve cells and glial cells in the frontal cortex of the control group (Figure 4A). The alcohol-treated group (Group II) showed a positive immune-reaction to caspase-8 (seen as a dark brown reaction) in the cytoplasm of many nerve cells and glial cells (Figure 4B). The ATOR-treated group (Group III) and the alcoholic group treated with ATOR (Group IV) showed a significant decrease in the percentage area of caspase-8 expression in the nerve cells and glial cells compared to the alcoholic group (Group II). While they showed a significant increase as compared to the control group (Group I) and the alcoholic group treated with ATOR and ASA (Group V), they did, however, show a non-significant change when compared to each other (Figure 4C,D, respectively). In contrast, the alcoholic group treated with the ATOR and ASA (Group V) showed a minimal or negative (in some fields) immune-reaction to caspase-8 in both the nerve cells and glial cells. Indeed, Group V statistically showed a non-significant change compared to the control group, but it showed a significant decrease when compared to all other groups (Figure 4E) (Table 3). These results indicate that the treatment with alcohol and/or ATOR significantly increased the expression of caspase-8, and that the ASA treatment was able to diminish caspase-8 expression in the nerve cells and glial cells of the frontal cortex.

#### 3.3.3. Semi-Thin Sections Analysis

**Figure 5:**
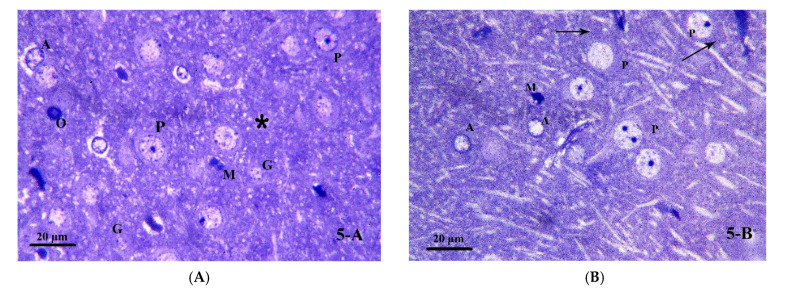

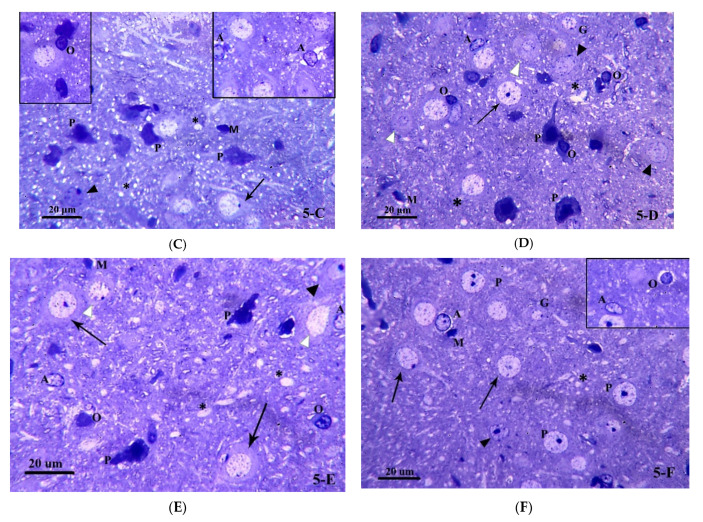
Photomicrograph of brain tissue semi-thin sections (toluidine blue stained) for (**A**) Group I, showing pyramidal cells (P) with large, rounded nuclei and prominent nucleoli. Granule cells (G) have small rounded euchromatic nuclei. Notice the presence of astrocytes (A), oligodendrocyte (O) and microglia (M). The neuropil is homogenous (*). (**B**) Group I, showing pyramidal cells (P) with large, rounded nuclei and prominent nucleoli. Nissl granules (↑) are seen in their cytoplasm. Notice the presence of astrocytes (A) and microglia (M). (**C**) Group II, showing most of the pyramidal cells (P) are shrunken, condensed and deeply stained. Few pyramidal cells are seen with large, rounded nuclei but with absent Nissl granules (↑). Others have large rounded dark nuclei (▲). Notice the presence of astrocytes (A) with irregular nuclei and surrounded by vacuoles, oligodendrocyte (O) and microglia (M). The neuropil is vacuolated (*). (**D**) Group III, demonstrating some pyramidal cells are shrunken, condensed and deeply stained (P). Some are seen with dark nuclei (▲) while others have large, rounded nuclei but with absent Nissl granules (↑). Some granule cells (G) have small rounded euchromatic nuclei while others have darkly stained nuclei (∆). Notice the presence of astrocytes (A), oligodendrocyte (O) and microglia (M). The neuropil is vacuolated (*). (**E**) Group IV, showing some pyramidal cells are shrunken, condensed and deeply stained (P). Some are seen with dark nuclei (▲) Some others are seen with pale, irregular nuclei (∆) while others have large, rounded nuclei but with absent Nissl granules (↑). Notice the presence of astrocytes (A), oligodendrocyte (O) and microglia (M). Notice the large vacuoles in the neuropil (*). (**F**) Group V, showing most of the pyramidal cells have large, rounded nuclei and prominent nucleoli (P). Notice the presence of Nissl granules (↑). A pyramidal cell with dark irregular nucleus is present (▲). Granule cells (G) have small rounded euchromatic nuclei. Notice the presence of astrocytes (A), oligodendrocyte (O) and microglia (M). Small vacuoles are seen in the neuropil (*). (toluidine blue × 1000).

Next, we performed semi-thin sections analysis on the brain tissue in order to explore the effect of chronic alcohol treatment and/or ATOR intake and the possible protective effect of ASA on the cells which are involved in the process of cognitive impairment and neuroinflammation. Examination of the semi-thin sections of the frontal cortex for the control group (Group I) showed pyramidal cells with large rounded vesicular nuclei and prominent nucleoli. Nissl granules were seen in their cytoplasm. Granule cells were seen with small rounded euchromatic nuclei surrounded by a rim of cytoplasm. Different types of glial cells were seen with their characteristic features: the astrocytes had large, pale nuclei with a dense rim of peripheral heterochromatin; the oligodendrocytes had rounded or oval, darkly stained nuclei; while the microglia had small, irregular, dense nuclei. The neuropil appeared homogenous (Figure 5A,B). In the alcohol treated group (Group II), most of the pyramidal cells were shrunken, condensed, and deeply stained. Few pyramidal cells had large, rounded nuclei but with absent Nissl granules. Others had large, rounded, dark nuclei. Astrocytes were seen with irregular nuclei and surrounded by vacuoles. Oligodendrocytes and microglia were seen. The neuropil was seen vacuolated (Figure 5C). In the ATOR treated group (Group III), some pyramidal cells were seen shrunken, condensed and deeply stained. Some had large darkly stained nuclei while others had large rounded vesicular nuclei but with absent Nissl granules. Some granule cells had small rounded euchromatic nuclei, while others had darkly stained nuclei. Astrocytes, oligodendrocyte and microglia were seen. The neuropil was vacuolated (Figure 5D). The alcoholic group treated with ATOR (Group IV) showed some shrunken, condensed and deeply stained pyramidal cells. Some had large darkly stained nuclei. Some others were seen with pale, irregular nuclei, while others had large, rounded nuclei but with absent Nissl granules. Astrocytes, oligodendrocyte and microglia were also seen. Large vacuoles were seen in the neuropil (Figure 5E). In the alcoholic group treated with the ATOR and ASA (Group V), most of the pyramidal cells showed large, rounded nuclei and prominent nucleoli. Nissl granules were seen in their cytoplasm. Interestingly, pyramidal cells with dark irregular nuclei were seen. Granule cells had small rounded euchromatic nuclei. Astrocytes, oligodendrocyte and microglia were present. Small vacuoles were seen in the neuropil (Figure 5F). These results indicate that treatment with alcohol and/or ATOR caused extensive damages to the microglial cells and astrocyte cells, while administration of the ASA could have attenuated these damages and returned the cells to almost the normal state.

#### 3.3.4. Transmission Electron Microscopic (TEM) Study

**Figure 6:**
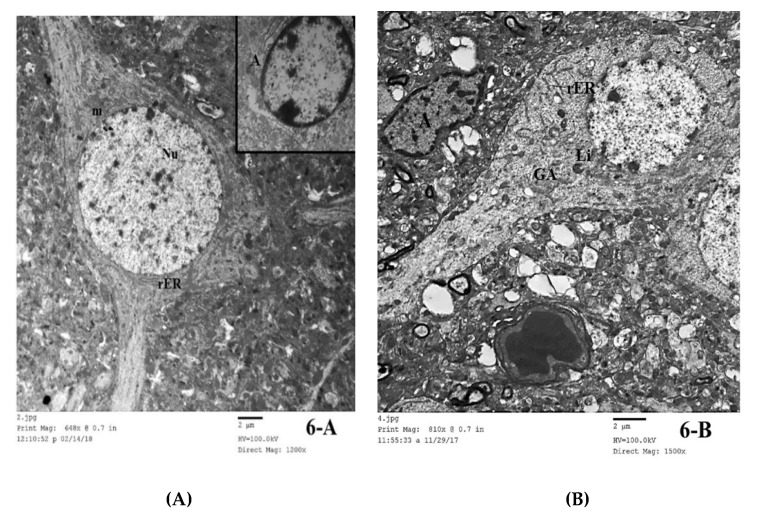

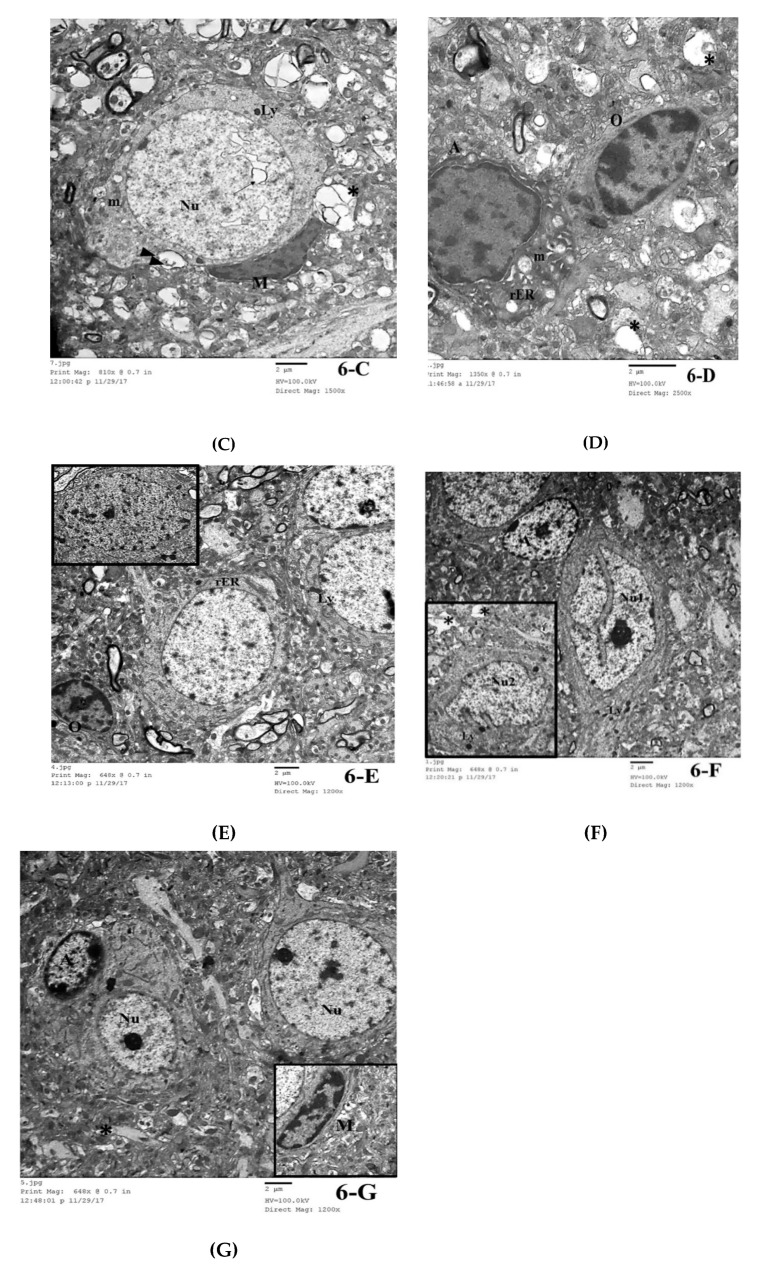
Electron microscopic examination (TEM) of frontal cortex in brain tissue for (**A**) Group I, showing a pyramidal cell in cerebral cortex of Group I. A large, rounded, central and euchromatic nucleus (Nu) is seen surrounded by cytoplasm. Notice the rough endoplasmic reticulum (rER) and mitochondria (m). Inset showing an astrocyte (A) Inset (TEM × 2000). (**B**) Group I, demonstrating a nerve cell with dilated, irregularly arranged cisternae of rough endoplasmic reticulum (rER). Notice the presence of lysosomes (Ly), vacuoles (↑) and a Golgi apparatus (GA) in the nerve cell. Notice the presence of an astrocyte (A) with irregular dark nucleus. Large vacuoles (*) are seen in the neuropil (TEM × 1500). (**C**) Group II, showing a nerve cell in the frontal cortex of Group II with large, rounded, central and euchromatic nucleus (Nu). Notice the intranuclear vacuoles (↑). Lysosomes (Ly), elongated mitochondria (m) and cytoplasmic vacuoles are seen. Notice a microglial cell is present beside the nerve cell. Many large vacuoles are seen in the neuropil (*) (TEM × 1500). (**D**) Group II, showing two glial cells in the frontal cortex of Group II. An astrocyte (A) is seen with an irregular dark nucleus and a dense rim of peripheral heterochromatin. Notice the dilated and distorted cisternae of rough endoplasmic reticulum (rER), mitochondria (m) with destroyed cristae and cytoplasmic vacuoles (↑). An oligodendrocyte (O) is seen with its dark oval nucleus and large clumps of heterochromatin. Multiple dense bodies are seen in its cytoplasm (▲). Large vacuoles are seen in the neuropil (*) (TEM × 2500). (**E**) Group III, showing pyramidal cells in the frontal cortex of Group III with large, rounded, central and euchromatic nuclei (Nu). Notice mild dilatation of the rough endoplasmic reticulum (rER). Lysosomes are seen (Ly). An oligodendrocyte (O) is present beside a nerve cell. Inset: showing a pyramidal cell with darkly stained irregular nucleus (TEM × 1200). (**F**) Group IV, showing a pyramidal cell in frontal cortex with marked deep indentation of the nuclear membrane (Nu1). Many lysosomes are seen (Ly). An astrocyte (A) is present beside the nerve cells. Inset: showing a pyramidal cell with indented and irregular nucleus (Nu2). Large vacuoles are seen in the neuropil (*) (TEM × 1200). (**G**) Group V, showing pyramidal cells in the frontal cortex of Group V with large, rounded, central and euchromatic nuclei (Nu). Notice mild dilatation of a few rough endoplasmic reticulum cisternae (rER). Few lysosomes are seen (Ly). An astrocyte (A) is present beside a nerve cell. The neuropil is homogenous (*). Inset: showing a microglial cell (M) (TEM × 1200).

To gain deep insight into our previous histological results and to explore the possible protective effect of ASA on brain tissue upon chronic alcohol abuse and/or ATOR treatment, extensive transmission electron microscopic examinations of ultra-thin sections of the frontal cortex were performed. TEM analysis for the control group showed pyramidal cells that were large and branched, with large, rounded, central and euchromatic nuclei surrounded by cytoplasm. The cytoplasm contained the usual organelles as rough endoplasmic reticulum (rER), mitochondria (M) and lysosomes (Ly). Astrocytes were seen with rounded euchromatic nuclei and a dense rim of peripheral heterochromatin (Figure 6A). In the alcohol treated group, some nerve cells showed large, rounded, central, euchromatic nuclei with intranuclear vacuoles. Their cytoplasm showed dilated, irregularly arranged cisternae of rER, elongated mitochondria, many lysosomes, and cytoplasmic vacuoles. Some astrocytes showed irregular dark nuclei and a dense rim of peripheral heterochromatin. They had dilated and distorted cisternae of the rER, mitochondria with destroyed cristae and cytoplasmic vacuoles. Oligodendrocyte were seen with their dark oval nuclei and large clumps of heterochromatin. Multiple dense bodies were seen in their cytoplasm. Large vacuoles were seen in the neuropil (Figure 6B–D). In the frontal cortex of the ATOR treated group, many pyramidal cells were seen comparable to the control group except for mild dilatation of the rER and presence of many lysosomes; however, some pyramidal cells appeared with darkly stained irregular nuclei. Oligodendrocytes were seen with their characteristic appearance (Figure 6E). In the alcoholic group treated with ATOR, many pyramidal cells showed marked deep indentations and irregularity of their nuclear membranes. Many lysosomes were seen in their cytoplasm. Astrocytes were seen with their characteristic appearance and large vacuoles were seen in the neuropil (Figure 6F). The alcoholic group treated with ATOR and ASA showed that most of the pyramidal cells appeared comparable to the control group. Only a few cisternae of the rER were mildly dilated and few lysosomes were seen. Astrocytes and microglial cells were seen with their characteristic appearance. The neuropil appeared homogenous (Figure 6G). These results reveal that there were several changes in the ultrastructure of the frontal cortex sections that occurred upon treatment with alcohol and/ATOR, and that the ASA administration could have diminished these changes and returned the cells nearly to the normal state.

### 3.4. In Silico Molecular Docking Study

**Figure 7:**
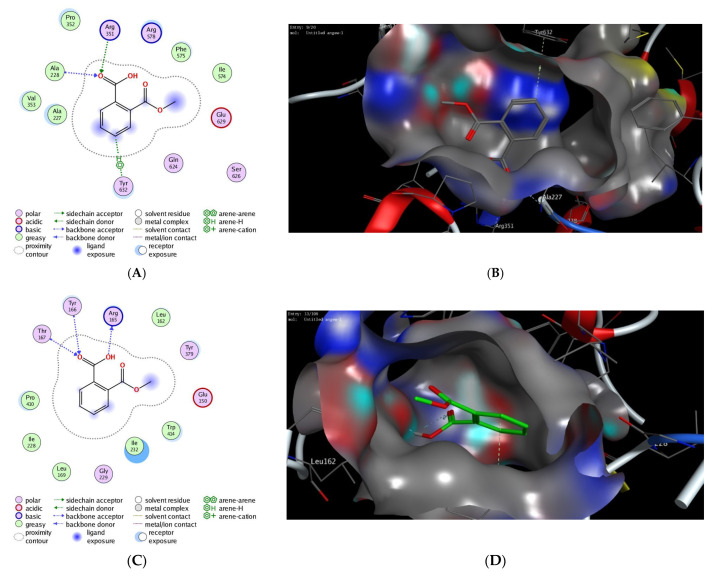
The 2D and 3D molecular modelling interactions of ASA (green and grey in 3D interactions) with NLRP3 protein: *7alv* (**A**,**B**), and *6npy* (**C**,**D**). Dotted green arrows represent the hydrogen bonds; (C atoms are colored green, S yellow and O red).

In silico molecular modelling represents a modern computational analysis that can be applied in investigating the possible mode of action for a drug [47]. To further explore the possibility of ASA to target the NLRP3 protein, we performed an extensive in silico molecular docking study to investigate the binding affinity of ASA into the active sites of the binding pockets of the NLRP3 protein. In the protein data bank, there were only two PDB codes available for NLRP3 proteins (PDB codes, *n6py* and *7alv*) which represent the 3D structure of theNLRP3 NACHT domain co-crystallized with a potent inhibitor (NP3-146) and/or ADP [39,40]. To gain insights into the different possible modes of binding for ASA, we performed molecular docking studies for both 3D crystal structures available (PDB codes, *n6py* and *7alv*), and the obtained results were isolated to be evaluated (Table 4). The obtained poses were evaluated and selected based upon the capability of the docked ligand to possess the main binding interactions to the active site of the binding protein. After adjusting different parameters, we examined our docking protocol by redocking the co-crystallized ligand, either ADP or NP3-146, to the active sites of the NLRP3 NACHT domain to ensure that the re-docked ligand could form the main interactions reported in the database. Next, the validated docking protocol was used to investigate and to assess the binding affinity of ASA to the ADP-binding site and the inhibitory-binding site of the NLRP3 NACHT domain. The obtained results from the docking study, docking score and observed binding interactions, are presented in Table 4. Based on the recently reported crystal structure, the NLRP3 NACHT domain had two binding sites: an ADP-binding site and inhibitory-binding site [39,40]. Our results revealed that the ASA had high binding scores to both ADP- and inhibitor-binding pockets with the ability to bind to the active sites of the NLRP3 NACHT domain by both hydrophilic and hydrophobic interactions in a similar mode compared to the original co-crystallized ligands (Figure 7, Table 4). These results indicate that the suppression effect of ASA on cognitive dysfunction could be also attributed to its ability to target the NLRP3 protein.

## 4. Discussion

Cognitive dysfunction is a common and potentially severe consequence of long-term alcohol abuse [48]. Continuous consumption of alcohol in high levels can lead to long-term functional changes such as impairments in visuo-spatial functioning [49]. Functional impairments in alcohol abuse can be explained by the changes in grey matter structure, resulting from the neurotoxicity of alcohol in chronic high consumption patterns [50]. Several studies have showed that in alcohol abuse patients the grey matter volume reduced as compared to healthy controls. Yang et al. reported several alterations in the grey matter in left and right precentral gyri, as well as in subcortical regions like the left thalamus and right hippocampus [51].

Alcohol abuse induces brain neurodegeneration resulting in elevation of proinflammatory cytokines and chemokines expression and, in particular, microglia can be activated through their receptors (such as Toll-like receptor 4) [52]. Robust changes in miRNA expression have been reported in the prefrontal cortex of subjects with a history of chronic alcohol abuse [17], which may provide the opportunity to evaluate ongoing changes in the CNS upon the initiation of neurodegeneration [53]. Among the different miRNA, miRNA155 is highly expressed in numerous tissues, including the brain, suggesting its pro-inflammatory action in CNS. miRNA155 induces neuroinflammation through a reduction in the endogenous anti-inflammatory response resulting in increased inflammation [54]. Neuroinflammation has been identified as a causative factor of multiple neurodegenerative diseases. The NLRP3 inflammasome—a subcellular multiprotein complex that is abundantly expressed in the CNS—can be activated by a wide range of exogenous and endogenous stimuli [55]. Activation of the NLRP3 inflammasomes pathway is responsible for neuroinflammation and is associated with several brain diseases [56]. Toll-like receptors (TLRs) in the CNS trigger the activation of pro-IL-1β and pro-IL-18 that are converted into their active forms by the NLRP3 inflammasome [57].

Alcoholic fatty liver (steatosis), alcoholic hepatitis, and fibrosis are associated with the progression of liver cirrhosis, with the highest risk in alcoholism [5]. Statins are lipid-lowering drugs that are utilized to treat lipid disorders and commonly prescribed for patients with fatty liver diseases to reduce low-density lipoprotein cholesterol [8]. The exact effect of statins on cognitive function has been not fully addressed; however, it was suggested that statins may cause a short-term cognitive impairment [58,59]. Cholesterol is a crucial lipid for brain functions, and it is used in the formation of the nervous system as well as the manufacture of steroid hormones that are involved in brain signaling. Accordingly, decreasing the serum cholesterol level, upon statins treatment, may affect cognition function [25]. Interestingly, it was suggested that switching from lipophilic statins (e.g., ATOR) to hydrophilic statins could resolve the cognitive impairment with vascular benefits [60].

Based on the abovementioned facts, in the present study we aimed at investigating the molecular and cellular effects of ATOR on cognitive dysfunction induced by alcohol intake. Accordingly, rats were treated with alcohol and/or ATOR and a set of biochemical, histopathological, and immunohistochemical analyses were performed. A Morris water maze test showed that the cognitive function was significantly impaired, as indicated by the increase in latency time to reach the target quadrant and the decrease in the % time spent in the target quadrant. Our results were in accordance with the previous findings which reported a significant impairment in the Morris water maze test for rats treated with alcohol, together with an impairment in the memory as demonstrated by the markedly increased latency time [61,62]. Further studies have showed that alcohol intake causes various neurological diseases via the toxic effect of various key mediators on the brain, such as inflammation, caspase-3, and the brain-derived neurotrophic factor BDNF [63]. Additionally, Stragier et al. reported that ethanol treatment in C57BL/6J mice causes profound impairment in the learning and memory place [64]. Toward the effect of statins on cognitive function, in accordance with our results, King et al. reported that statin therapy significantly and temporally impairs cognitive function and recommended that clinicians should be aware of cognitive impairment as a potential adverse effect associated with statin therapy [65].

As the alcohol induced neuroinflammation in the brain, the IL-1β level increased through the activation of the NLRP3 inflammasome [66]. Our results demonstrated a pivotal role for NLRP3 inflammasome activation that mediates the IL-1β level in the alcohol-related neuroinflammation. In agreement with Orio et al., we found that treatment with alcohol or in combination with ATOR produced a significant increase in caspase-8 and the IL1β and NLRP3 inflammasomes in the brain tissue [67]. Indeed, Orio et al. showed that alcohol abuse causes oxidative damage to the mitochondria and cellular proteins which leads to the development of neuroinflammation and neurological disorders. Further, the inflammasome components (NLRP1, NLRP3, ASC) and proinflammatory cytokines (TNF-α) levels were increased in alcohol-treated brains, together with an enhancement of caspase-1 activity and the IL-1β protein [21]. Our results revealed that the ATOR increased the expression of IL-1β, caspase-8 and the NLRP3 inflammasome pathway. Statins possess anti-inflammatory effects which lead to enhancing the activity of NLRP3 inflammasome and IL-1β [68,69]. According to Peng et al. and Shu et al., ATOR has an antioxidant capacity via increasing the level of GSSG/GSH, and is considered as an anti-inflammatory agent and proinflammatory compound [70,71].

In the present study, the treatment with alcohol alone or in combination with ATOR produced a significant increase in the expression of miRNA155 levels in the brain tissue. Although previous studies have confirmed the relation between cognitive dysfunction and miRAN155 expression, the link between miRNA155 expression and alcohol-induced cognitive dysfunction was unique in our study. In our study, we hypothesized that the link between alcohol-induced cognitive dysfunction and miRNA155 could be via the NLRP3 inflammasome pathway. This hypothesis was also supported by the recent findings by Liu et al. which showed that miRNA155 expression increases in the hippocampus of AD rats, together with IL-1β, IL-6 and TNF-α [18]. Our findings were in agreement with Yin et al.’s study which showed that miRNA155 induced the expression of NLRP3 inflammasome which could induce the secretion of IL-1β and IL-18 leading to the development of inflammation and atherosclerosis [72]. Silencing of miRNA155 was shown to significantly decrease the inflammation and NLRP3/caspase-1 signaling [73].

As mentioned above, the brain tissue was affected by alcohol and/or ATOR administration leading to neuroinflammation. Our results were further confirmed by light microscopic examination of the brain tissue. In the control group, the frontal cortex showed a normal appearance with the six layers of the cortex formed of neurons and glial cells, as previously described [74]. On the other hand, the groups treated with alcohol, ATOR or a combination of both showed signs of degeneration in the neurons as well as the glial cells, in the form of shrunken cells surrounded by hallows, a loss of Nissl’s granules (seen in the semi-thin sections) and deeply stained, pyknotic nuclei. Interestingly, the alcoholic group showed aggregations of inflammatory cells—most probably microglia—forming a granuloma-like structure. These lesions were previously shown to be associated with the degeneration of neurons that was caused due to the consumption of the alcohol and/or ATOR [75]. Moreover, the present morphometric study showed a significant decrease in the mean thickness of the frontal cortex in the alcoholic group as compared to the control group. These results were in accordance with an Olawale et al. study which showed that cerebral cortex damage, due to acute alcohol intake, is associated with oxidative stress [76]. Fortier et al. reported that the thickness of the brain cortex significantly decreased in specific areas in alcoholic compared with non-alcoholic members, suggesting that alcohol may affect the large pyramidal cells in the frontal cortex [77]. Moreover, Saito et al. reported changes in the count of glial cells after chronic ethanol exposure [52]. Together, these findings indicate that alcohol consumption causes a decline in grey matter size in certain brain regions and has effects on the behavioral depiction of these brain areas that may lead to cognitive impairment [50].

Caspase-8 plays a central role in apoptosis, as well as regulating inflammation by modulating IL-1β expression. In the present study, examination of caspase-8 stained brain sections of the control group showed a negative reaction. While the alcoholic group and ATOR-treated alcoholic group showed a positive reaction to caspase-8, in both the nerve cells and the glial cells. Previous co-immunoprecipitation studies demonstrated that caspase-8 presents in the NLRP3 inflammasome complex, where it is believed to be involved in the cleavage of pro-caspase-1 [78]. Further, different inflammatory stimuli can cause activation of NF-kB via the association of caspase-8 [79,80]. These results could explain the observed increased caspase-8 in our investigations.

Our findings were further confirmed by a TEM study which revealed neuronal and glial damage in the alcoholic group. Damaged neurons showed dilated, irregularly arranged cisternae of the rER, elongated mitochondria, many lysosomes and cytoplasmic vacuoles. Similar changes were previously described [81]. Some astrocytes showed irregular dark nuclei and a dense rim of peripheral heterochromatin. They had dilated and distorted cisternae of the rER, mitochondria with destroyed cristae and cytoplasmic vacuoles. A similar pattern of rER was previously observed in hepatocytes due to chronic alcohol consumption, and was attributed to endoplasmic reticulum stress [28]. In the present study, treatment with alcohol and/or ATOR caused nearly similar effects on the neurons structure and glial cells in the frontal lobe of the brain tissue.

**Figure 8:**
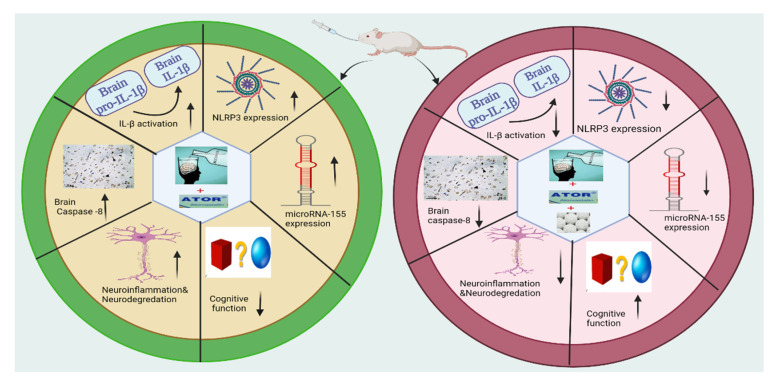
Molecular and cellular effects of ASA on cognitive dysfunction induced by alcohol and/or ATOR intake.

ASA, as a nonsteroidal anti-inflammatory, is frequently used as a potent drug in several diseases. The anti-inflammatory activity of ASA is derived from its ability to inhibit cyclooxygenase which suppresses the production of pro-inflammatory molecules, such as prostaglandins [82]. Several studies have demonstrated the capability of ASA to attenuate the hepatotoxicity and endothelial dysfunction by regulating the NLRP3 inflammasome and IL-1β levels [26,28,29]. These pleiotropic modes of action suggest that ASA could be utilized in alcoholism for the treatment of cognitive dysfunction. Toward this aim, we have investigated the efficacy of ASA to treat the alcohol-induced cognitive impairment associated with ATOR intake (Figure 8). In our investigations, we reported that treatment with ASA significantly reduced the expression of caspase-8, NLRP3, and IL-1β levels in brain tissue. These results could be attributed to the anti-inflammatory properties of ASA which could change the innate immune response and autophagy via the elimination of impaired mitochondria and oxidative stress [83,84]. Further, it was shown that ASA administration decreased the expression of miRNA155 in brain tissue. Taken together, our results indicated that ASA treatment has suppression effects on several molecular factors of cognitive dysfunction. These findings were further confirmed by the histopathological studies which revealed a regular arrangement of the cerebral cortex layers with noticeable improvement in the structure of both neurons and glial cells in brain tissue.

Finally, an extensive molecular modelling study was performed to investigate the binding affinity of ASA to the binding pockets of the NLRP3 NACHT domain. Recent examination of the 3D structures of the NLRP3 NACHT domain revealed that the protein has two binding sites: an ADP-binding pocket and a small inhibitor-stimulate-binding pocket [39,40]. The ADP-binding pocket revealed a set of key amino acid residues (Thr231, Ile232, Gly239, Thr167, Tyr166, His520, Lys 230) that interacted with one molecule of ADP to form a stable network of hydrophilic interactions. While the recently discovered inhibitor-binding pocket was shown to possess a slightly basic active site (Arg351, Arg578, Pro352, Ala228) which mainly binds to the sulfonylurea part of the co-crystallized inhibitor (NP3-146). As shown in Table 4, the ASA demonstrated high negatively-binding scores toward the binding pockets of the NLRP3 NACHT domain, indicating that the binding of ASA to the active sites of NLRP3 is thermodynamically favorable. Toward the ADP-binding pocket, the ASA showed the ability to bind to the active site through interaction of the carboxylic moiety to two of the main active amino acid residues (Ala228, Tyr632), and an additional basic amino acid residue (Arg351). This interaction was also supported by hydrophobic interactions with a set of greasy amino acid residues. Similarly, the ASA showed the possibility to bind to the active site of the inhibitor-binding pocket via a hydrophilic interaction with three of the main amino acid residues (Ala228, Arg351, Tyr632) together with a set of hydrophobic interactions with greasy amino acid residues. Taken together, our molecular docking study findings indicate that the suppression effect of ASA on cognitive dysfunction could be attributed to its ability to target the active sites of the NLRP3 NACHT domain. Further investigation should be performed in future to deeply investigate the inhibitory activity of ASA toward the NLRP3 protein which would highlight ASA as a possible drug for cognitive dysfunction.

## 5. Conclusions

In the present study, we showed that alcohol intake either alone or in combination with ATOR caused changes in the biochemical, immunohistochemical and histological parameters of brain tissue. Our study revealed that administration of alcohol either alone or in combination with ATOR caused an upregulation of NLRP3/IL1B inflammasome and its epigenetic regulator, miRNA155, suggesting a key role in neuroinflammation and neurodegeneration. The histological and morphometric analysis indicated neuronal and glial cell damage as well as aggregations of microglial cells in brain tissue. On the other hand, ASA administration caused a significant downregulation of the miRNA155/NLRP3/IL1B inflammasome pathway. The protective effect of ASA was confirmed by histopathological, morphological and immunohistochemistry studies which revealed a decrease in neuronal damage and diminished microglial cell infiltration in brain tissue with a decrease in caspase-8 immunoreaction in neurons and glial cells. Finally, our molecular modelling study indicated the ability of ASA to bind to the binding pockets of the NLRP3 NACHT domain with high binding scores. Collectively, our findings demonstrated that ASA administration has beneficial and protective effects on cognitive function. Accordingly, we hypothesize that the administration of ASA to patients with alcoholism and/or long-term ATOR treatment could effectively attenuate the progression of cognitive impairment.

## Figures and Tables

**Table 1:** Assessment of cognitive function in the different treated groups by the Morris water maze (MWM) test (mean ± SD).

Groups Latency to Reach Target Quadrant (s)	Day 1	Day 2	Day 3	Day 4	Day 5	Day 6	% Time Spent in Target Quadrant
Control group	6.68 ± 19.9	27.88 ± 6.6	22.18 ± 8.1	15.3 ± 7.5	8.37 ± 2.5	7.7 ± 4.9	47.33 ± 7.5
Alcohol group	10.9 ± 9.9 ^a^	67.63 ± 5.4 ^a^	92.9 ± 7.1 ^a^	55.52 ± 6.3 ^a^	45.38 ± 8.2 ^a^	21.08 ± 7.2 ^a^	19.25 ± 5.9 ^a^
ATOR group	8.06 ± 17.2 ^a^	50.77 ± 10.2 ^a^	80.9 ± 9.8 ^a^	32.6 ± 5.8 ^a^	35.33 ± 13.3 ^a^	19.17 ± 6.4 ^a^	21.6 ± 10.3 ^a^
Alcohol + ATOR group	13.71 ± 7.3 ^a^	85.19 ± 8.8 ^a^	71.46 ± 7.7 ^a^	48.23 ± 13.2 ^a^	55.5 ± 11.9 ^a^	33.17 ± 9.2 ^a^	12.81 ± 14.9 ^a^
Alcohol + ATOR + ASA group	7.73 ± 16.6 ^abcd^	33.1 ± 15.1 ^abcd^	32.23 ± 7.1 ^abcd^	21.17 ± 11.4 ^abcd^	21.9 ± 11.0 ^abcd^	15.08 ± 5.2 ^abcd^	36.6 ± 18.2 ^bcd^

Data are mean ± SD of 6 rats per group. *p* < 0.05 is significant; ^a^ *P* versus control group, ^b^ *P* versus Alcohol group, ^c^ *P* versus ATOR group, ^d^ *p* versus Alcohol + ATOR + ASA group.

**Table 2:** Effect of alcohol and/or ATOR on mean thickness of frontal cortex and assessment of ASA treatment (mean ± SD).

Group	Mean Thickness of Frontal Cortex
Group I	1832.15 ± 198.5
Group II	1455.60 ± 78.3 ^a^
Group III	1644.70 ± 185.2 ^b^
Group IV	1554.16 ± 69.8 ^c^
Group V	1800.70 ± 167.3 ^d^

^a^ Significant decrease compared to groups I, III and V, non-significant change compared to IV. ^b^ Significant change compared to groups, I, II and V, non-significant change compared to IV. ^c^ Significant change compared to groups I and V, non-significant change compared to II, III. ^d^ Significant increase compared to groups II, III and IV, non-significant change compared to I.

**Table 3:** Effect of alcohol and/or ATOR treatment on caspase-8 immune reaction and the influence of ASA administration (mean ± SD).

Group	Area % of Caspase-8 Positive Immune Reaction
Group I	0
Group II	4.41 ± 0.5 ^a^
Group III	1.75 ± 0.3 ^b^
Group IV	2.73 ± 0.4 ^c^
Group V	0.42 ± 0.1 ^d^

^a^ Significant increase compared to all groups. ^b^ Significant change compared to groups I, II and V and non-significant change compared to IV. ^c^ Significant change compared to groups I, II and V and non-significant change compared to III. ^d^ Significant decrease compared to groups II, III and IV and non-significant change compared to I.

**Table 4:** Scores (kcal/mol) and interactions of the molecular modelling process of ASA and ATOR drugs in the NLRP3 binding sites.

PDB	Docking Score (kcal/mol)	Interactive Residues	
		Hydrophilic Interactions	Hydrophobic Interactions
*7alv*	−12.17	Ala228, Arg351, Tyr632	Val353, Ile574, Phe575, Pro352, Ala227
*6npy*	−11.68	Arg165, Thr167, Tyr166	Leu162, Ile228, Trp414, Leu169, Pro410, Ile232

## Data Availability

Not applicable.
